# High resolution low kV EBSD of heavily deformed and nanocrystalline Aluminium by dictionary-based indexing

**DOI:** 10.1038/s41598-018-29315-8

**Published:** 2018-07-20

**Authors:** Saransh Singh, Yi Guo, Bartłomiej Winiarski, Timothy L. Burnett, Philip J. Withers, Marc De Graef

**Affiliations:** 1 Department of Materials Science and Engineering, Carnegie Mellon University, Pittsburgh, PA15213-3890 USA; 2 School of Materials, University of Manchester, Manchester, M13 9PL UK; 3 Thermo Fisher Scientific, Materials and Structural Analysis Division, Brno, 627 00 Czech Republic

## Abstract

We demonstrate the capability of a novel Electron Backscatter Diffraction (EBSD) dictionary indexing (DI) approach by means of orientation mapping of a highly deformed graded microstructure in a shot peened Aluminium 7075-T651 alloy. A low microscope accelerating voltage was used to extract, for the first time from a bulk sample, statistically significant orientation information from a region close to a shot crater, showing both recrystallized nano-grains and heavily deformed grains. We show that the robust nature of the DI method allows for faster acquisition of lower quality patterns, limited only by the camera hardware, compared to the acquisition speed and pattern quality required for the conventional Hough indexing (HI) approach. The proposed method paves the way for the quantitative and accurate EBSD characterization of heavily deformed microstructures at a sub-micrometer length scale in cases where the current indexing techniques largely fail.

## Introduction

The texture of a material, i.e., the statistical distribution of the 3D orientations of the crystallites in that material, is of central importance to the ultimate properties and performance of an engineering component. The orientation distribution function (ODF) of a polycrystalline material has traditionally been measured using X-ray diffraction techniques, which provide an average over many grains but no local spatial information. Electron backscatter diffraction (EBSD), the primary tool for lattice orientation determinations, has filled that gap for the past 25 years, and has allowed us to understand recrystallization, accommodation of deformation, phase changes, etc. in geology and materials science. The conventional pattern indexing method^1^ involves performing a Hough transform of the pattern to extract linear features known as Kikuchi bands; each band corresponds to Bragg reflection by a set of crystallographic planes. The grain orientation is then assigned by comparing the relative band arrangement to a pre-computed table of interplanar angles. One of the primary methods for improving the spatial resolution of EBSD is to lower the microscope accelerating voltage, which reduces the interaction volume, but has a detrimental effect on the pattern quality, rendering the indexing process more difficult. A completely new approach which fundamentally moves away from feature detection is required to overcome the difficulties in characterizing heavily deformed microstructures. In this paper, we achieve the necessary indexing fidelity at low acceleration voltage using the newly developed dictionary indexing (DI) approach (see methods section, Supplementary material and^2,3^) to examine the microstructure near the peen craters of a shot peened AA7075 T651 aluminium alloy^4^.

The complex microstructures generated by mechanical deformation processes span multiple length scales and represent challenging characterization problems. Among the many mechanical processes used to harden the surface of an engineering component and make it more fatigue resistant, the shot peening process entails shooting small hard spheres, e.g., fused ceramic beads, at the surface to induce a work-hardened compressive residual stress state in the near surface layers. Resolving the resulting microstructure requires a technique with a sufficiently high spatial and angular resolution to capture the dynamically recrystallized nanometer scale grains close to the shot peening surface, which is fast enough to gather microstructural data from a field of view in the ten to hundred micrometer scale. Currently, a combination of characterization modalities is used to analyze features at different length scales followed by a stitching or a statistical pooling of the data. There have been numerous studies to characterize the shot peened microstructure in a variety of material systems, including technologically significant Nickel based superalloys, Titanium alloys and steels^5–14^. However, none of these studies have been able to properly characterize the fine microstructure close to the shot crater and retain the context of these regions within the bulk specimen. In this paper, a new approach based on a combination of low keV EBSD alongside the newly developed dictionary indexing (DI) approach^2,3^ is investigated. It bridges the gap between length scales by providing both high spatial and angular resolution.

## Results

**Figure 1:**
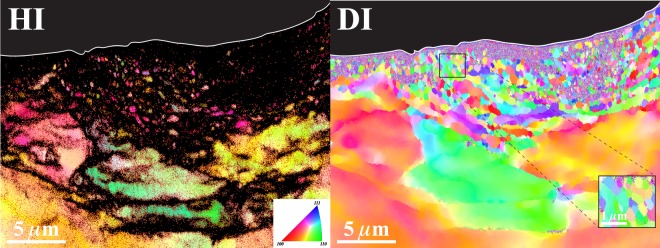
Comparison of traditional and dictionary indexing. Side-by-side comparison of orientation maps obtained by conventional Hough-based indexing (HI) and the new dictionary indexing (DI) of the same dataset. Colors (see stereographic projection triangle in the inset) indicate which crystal direction is normal to the sampling plane.

As explained in detail in the Methods section as well as in Fig. 1 of the Supplemental Information, a 10 kV EBSD scan was carried out on a sample plane cut normal through the base of a shot peen crater; the scan comprises approximately 3.4 million (1,931 × 1,784) EBSD patterns (EBSPs) over an area of 56 × 52 *μ*m^2^. The diffraction patterns were indexed using the Hough-based indexing (HI) technique as well as the new dictionary indexing (DI) technique. Figure 1 illustrates the power of this approach to produce a map from a dataset that cannot be analysed by the conventional approach in the form of inverse pole figures (IPFs) for the two indexing approaches for a portion of the complete scan. An inverse pole figure shows the spatial distribution of crystal lattice directions with respect to a selected sample direction, in this case a direction normal to the scan surface (see Supplemental Information Fig. 1). The shot peen crater surface is indicated by the white line near the top of the IPFs. It is clear from Fig. 1-HI that conventional Hough-based indexing is unable to reveal the near-surface nanocrystalline grains arising from the severe plastic deformation; all black pixels below the white line correspond to unindexed diffraction patterns. By contrast, after off-line processing of the raw Kikuchi pattern data using the DI approach, the nanocrystals near the shot peen crater are clearly visible in Fig. 1-DI. In addition, the color gradients across the larger regions in the center portion of the maps indicate that the lattice orientation changes by tens of degrees over a distance of a few tens of microns. The recrystallized grains near the crater outline, however, have mostly uniform coloring (see lower right inset), indicating that these grains are relatively strain-free.

**Figure 2:**
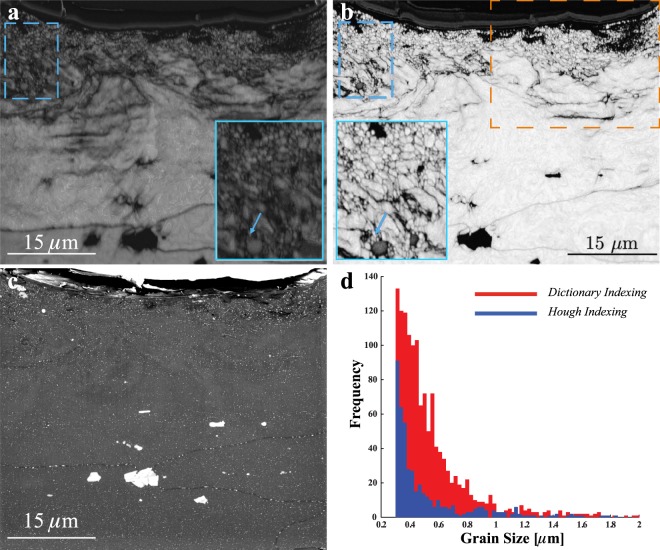
Grain size analysis in the nanocrystalline region. (**a**) Image quality map formed from the original EBSPs and (**b**) orientation similarity map (OSM) from the new DI approach. The insets in a and b are magnified views of the marked regions and arrows indicate nano-scale grains surrounding the second phase particle successfully reconstructed from DI. (**c**) shows a back scattered electron image revealing the secondary phase particles and a single large grain directly beneath the shot crater; (**d**) shows the grain size distribution of the Hough and Dictionary indexed data.

Figure 2a and b show the image quality map using the conventional Hough-based method (a) and the orientation similarity map (OSM, defined in the Methods section) obtained using the dictionary method (b). The blue outlined insets correspond to the region delineated by a blue hashed rectangle in the upper left corner of each image; the nanocrystalline grains indicated by the blue arrows appear fuzzy when analyzed with the Hough method, but are well defined in the OSM. This is expected since the image quality, as defined for the conventional method, measures the average height of the detected Hough peaks, and in the nanocrystalline region there are fewer detectable Hough peaks, thus reducing the image quality. The orientation similarity map, on the other hand, shows a nearly constant intensity inside grains, and a clear intensity decrease at grain boundaries; thus, the grain boundary network is more clearly delineated using the DI approach. The orange rectangle in the upper right of Fig. 2b delineates the region used to create the IPF maps of Fig. 1. In the Supplementary Information, additional maps generated using the dictionary method are available. Figure 2c shows the corresponding backscatter electron image of the scan region; the second phase particles appear bright on a darker background and several grain boundaries are visible, decorated with second phase particles. The grain size distributions calculated using the conventional approach and the dictionary method are shown in Fig. 2d; the Hough method fails to correctly index the smaller grains (<1 *μ*m), resulting in an underestimation of the number of smaller grains in the recrystallized near surface zone.

**Figure 3:**
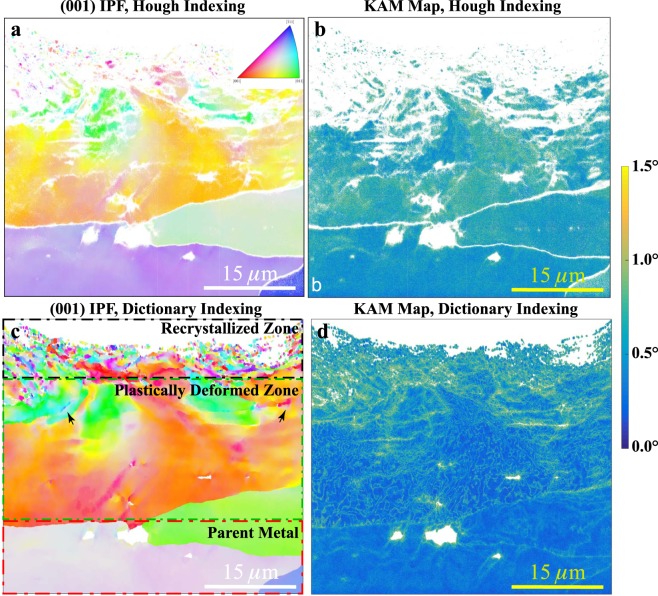
Inverse pole figure and kernel average misorientation map comparisons. (**a**) and (**c**) respectively are the Hough and Dictionary indexed IPF (001) maps; (**b**) and (**d**) respectively are the kernel average misorientation (KAM) maps obtained from Hough indexing and dictionary indexing for a 1.5° maximum threshold.

Figure 3 shows the (001) inverse pole figure (IPF; legend as inset) of the indexed scan points (a) and the kernel average misorientation (KAM) map (b) obtained using the conventional indexing approach for a 1.5° threshold. Only a few of the grains close to the shot peened surface are indexed (unindexed points are represented in white); similarly, the indexing rate is very low in the highly deformed regions, the regions close to the second phase particles and near grain boundaries. The corresponding IPF map calculated using the dictionary approach is shown in Fig. 3c; all grains below a threshold size of 0.08 *μ*m have been removed (represented in white). The dictionary method not only captures the orientations of the highly deformed regions, the inter-phase and inter-grain boundaries, but also reveals the orientations of the nanocrystalline grains in exquisite detail. The KAM map generated using the dictionary approach is shown in Fig. 3d. Based on the IPF and KAM maps from the dictionary method, the deformed microstructure can be broadly classified into three distinct regions: a *recrystallized region* consisting of nano-grains extending to a depth of about 3–5 *μ*m, a *plastically deformed zone* extending to about 20–25 *μ*m which includes a great deal of stored plastic work, and finally the *parent metal zone* exhibiting low orientation gradients. Additionally, possible nucleation sites of new grains can also be seen in the highly deformed transition region, as indicated by the two arrows in Fig. 3c. These are completely absent from the HI indexing result due to the extreme deformation surrounding these regions.

**Figure 4:**
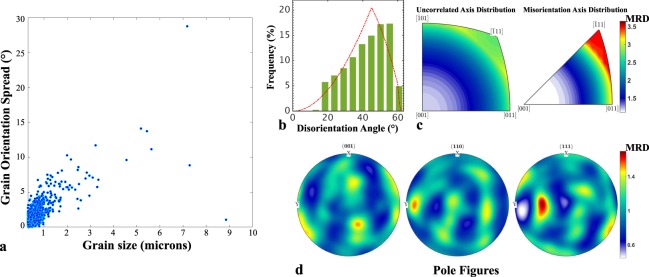
Grain orientation and grain boundary statistics. (**a**) Grain orientation spread map vs. equivalent grain size map; (**b**) and (**c**) grain boundary disorientation statistics and (**d**), (100), (110) and (111) pole figures for the texture in the recrystallized zone.

The large number of successfully indexed nano-sized grains using the dictionary method allows for a quantification of the local texture in the recrystallized zone. The grain orientation spread (GOS) vs. the equivalent grain size (grain size = $2\sqrt{{\rm{grain}}\,{\rm{area}}/\pi }$) map is shown in Fig. 4a. There is a correlation between the orientation spread, which can be interpreted as a proxy for the stored deformation, with the equivalent grain diameter. Smaller grains, which are dynamically recrystallized, show a low orientation spread whereas the larger grains store more deformation. Panels b and c Fig. 4 show the disorientation angle and axis distributions of the grain boundaries for the recrystallized zone along with the uncorrelated distributions. The disorientation angle distribution deviates slightly from the Mackenzie distribution^15^ with many of the grain boundaries having high angle character. Additionally, the (111) direction is the (slightly) favoured disorientation axis compared to the uncorrelated distribution with a peak intensity of approximately 3.5 MRD (Multiples of Random Distribution). Finally, Fig. 4d shows the (100), (110) and (111) pole figures for the orientations in the recrystallized zone, revealing an almost random texture with a peak intensity in the (111) pole of approximately 1.5 MRD.

**Figure 5:**
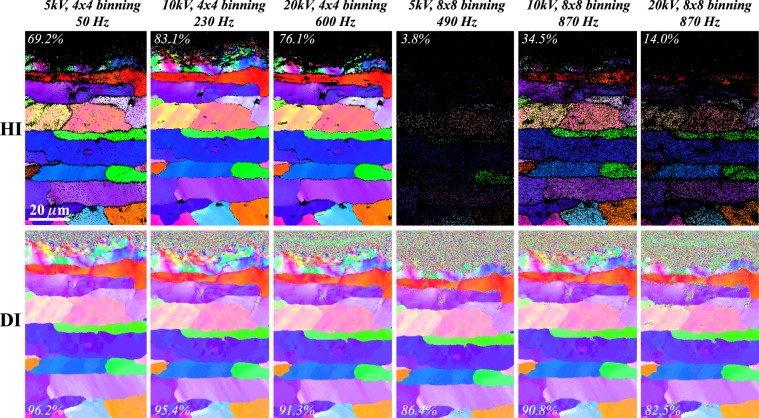
Comparison between Hough indexed and Dictionary indexed EBSD results under different acquisition conditions. The EBSD conditions and the resulting frame rates (number of patterns acquired per second) are labeled above each of the columns. The numbers in the insets represent measures for the indexing success rate for the highlighted rectangular box in the first map on the top row.

The potential of the dictionary-based indexing to extend the application of EBSD compared to the Hough-based approach can be further illustrated by considering the results from a comparative EBSD study for a number of different pattern acquisition conditions; six different data sets were acquired with different microscope accelerating voltages and pattern binning parameters, and the resulting (001) IPFs are shown in Fig. 5. Under extreme conditions, e.g., at 5 kV and 8 × 8 camera binning, a significant part of the heavily deformed zone as well as almost the entire unindexed parent metal zone are successfully indexed by the dictionary method. In addition, the dictionary indexed maps remain consistent over a large range of experimental conditions. These results highlight the possibility of imaging heavily deformed microstructures without significant acquisition time penalties. At the same time as being able to exploit DI for improved indexing it is also possible to exploit it for the benefit of speed of acquisition. Figure 5 shows that to extract similar quality maps (% indexed points), DI can operate at 10 kV with a step size of 120 nm utilizing 8 × 8 binning achieving a speed of 870 Hz, which is essentially hardware limited. In comparison, in order to achieve equivalent indexing, an acquisition speed of only 230 Hz is possible utilizing HI, resulting in an approximately 4× increase in acquisition speed. At 5 kV, the comparison is even more pronounced, providing a 10× speed up when using DI. The effect is less pronounced at 20 kV and although a hardware limit is reached, it is likely that the interaction volume, which is larger for higher accelerating voltages, plays a dominant role in limiting the indexing ability. The percentage numbers in Fig. 5 represent the indexing success rate for the field of view of each data set. For the HI results, the numbers indicate the percentage of patterns indexed with a mean angular deviation (MAD) below 1° (the mean angular deviation is defined in the Aztec 3.0 software as the angular deviation between the observed and simulated lattice plane orientations). For the DI results, the indexing success rate involves computation of the smallest disorientation between the top matching orientation and the next *M* nearest matches, where *M* was set to 5 in this case. If the smallest disorientation is smaller than a threshold value related to the angular step size used to sample orientation space, then the indexing is considered to be a success. For the sampling points at the top of the regions in Fig. 5, the set of *M* next best matches has nothing in common with the top match and hence the smallest disorientation angle is generally quite a bit larger than the threshold value, which was set to 1.5° for all data sets. The indexing success rate for the DI approach is significantly higher than that of the HI approach in all cases, in particular for the 8 × 8 binned data.

## Discussion

We have mapped the complex microstructure formed as a result of the shot peening of a 7075-T561 Aluminium alloy using a combination of low keV EBSD together with the Dictionary Indexing (DI) approach and an orientation refinement procedure. The results show a significant improvement over current commercially available EBSD software systems, with the ability to recover crystal orientations from nanocrystalline grains in the challenging low atomic number Aluminium alloy. The microstructure shows three distinct regimes: a dynamically recrystallized nanocrystalline zone, a plastically deformed transition zone with significant stored deformation and orientation gradients and the parent metal zone. The recrystallized zone shows an almost random texture with about 1.5 MRD peak value. The grain boundary disorientation angle and boundary axis distribution deviated somewhat from the random distributions, with a slight preference for high angle boundaries and the (111) boundary axis. The data reported here presents a significant challenge to the modelling community. It is expected that the improvements in the quality of microstructure characterization will spur interest in the quantitative modelling of the shot peening and other deformation processes, which produce such graded microstructures extending down to the nanoscale; we have shown that the DI approach is capable of providing quantitative data down to the sub-micrometer scale for direct comparison with deformation models.

Our results suggest that dictionary-based indexing can provide high quality EBSD analysis in situations where existing Hough-based methods fall short. Indeed, the DI approach provides maps of exquisite detail showing features that are not apparent in conventionally indexed maps. These include situations where the grain size is small, where the level of deformation is high, or where low quality signals are needed in order to acquire large areas quickly or avoid beam damage to the sample.

## Methods

### Sample preparation

The alloy under study is a 7075 Aluminium alloy double notch coupon^4^, in the T651 temper. For shot peening, fused ceramic beads (ZrO_2_ 67%, SiO_2_ 31%, size 63–125 *μ*m) were used, which were found to induce a higher fatigue strength improvement as compared to steel shots, without introducing undesired galvanic effects. An air-blast machine was utilized to perform the shot peening treatment with the following conditions: air pressure 1.0 bar, bead speed 57 m/s, coverage 100%, Almen intensity 4.5 N, nozzle 12 mm, angle of impingement 90°, working distance 100 mm, flow rate 5 kg/min.

The specimen used for this study consisted of a (250 × 150 × 150 *μ*m^3^) block and was the subject of a previous 3D-EBSD study^16^ which consumed part of the block. The remaining block face was freshly prepared at the shot-peened notch root for this study. The extraction of the block and the sectioning and polishing of the block face were conducted using a Thermo Fisher Scientific Helios^TM^ Plasma Xe^+^ FIB-SEM DualBeam equipped with an EasyLift^TM^ *in-situ* nanomanipulator on a TEM grid attached to a pre-tilted EBSD sample holder. The detailed procedure is presented in^16^, while the main steps comprising the lift-out procedure are summarized in Fig. 1 of the Supplementary Information. The block face was prepared at 30 kV and a current of 180 nA using an automated rocking polish routine implemented within Auto Slice and View 4.0 software^17^.

### Experimental details

EBSD scans were performed in a Helios Plasma FIB-SEM coupled with an Oxford Instrument NordlysNano EBSD detector and Aztec 3.0 software. EBSD data shown in Figs 1 and 3 was generated under 10 kV, 4 × 4 binning, and 25 nm step size. The EBSD data sets of Fig. 5 were generated at step sizes of 120 nm with accelerating voltages 5 kV, 10 kV and 20 kV. Both 4 × 4 and 8 × 8 camera binning were used for comparison. Single frame averaging was used and 10 lines at band centers and a Hough resolution of 75%. This was intended to simplify the indexing procedure and optimize speed. It is clear that there are many options for increasing the accuracy of the indexing but all of these come with a penalty of decreased acquisition speed. The exact same acquired data was indexed by the two different approaches.

### Dictionary-based indexing

To optimize the acquisition conditions for high spatial resolution while maintaining the fidelity of grain orientation reconstruction, the newly developed dictionary indexing (DI) approach^2^ was used. The efficacy of the method is rooted in an accurate physics-based forward model of the diffraction process. The dictionary indexing algorithm differs from the commercial Hough transform based method in the sense that no feature extraction is performed. Instead, the dictionary indexing method compares each experimental pattern with a dictionary of simulated diffraction patterns having different orientations, using the normalized dot product as the similarity metric. The orientation corresponding to the best match (i.e., the highest normalized dot product) is assigned as the orientation of the experimental pattern. Additionally, the list of top *k* matches can be retained and used to design supplementary microstructural descriptors. The Orientation Similarity (OS) is one such descriptor. This parameter measures the orientation similarity of a scan point with its nearest neighbors using the common elements in the list of top *k* matches and is an effective parameter to study intragranular orientation gradients as well as grain boundaries. For a scan point (*i*, *j*) in a rectangular scan, the OS, *η*_*i*,*j*_ is mathematically expressed as:

1 $${\eta }_{i,j}=\frac{1}{4}[\#({{\mathscr{S}}}_{i,j}\cap {{\mathscr{S}}}_{i-\mathrm{1,}j})+\,\#({{\mathscr{S}}}_{i,j}\cap {{\mathscr{S}}}_{i+\mathrm{1,}j})+\,\#({{\mathscr{S}}}_{i,j}\cap {{\mathscr{S}}}_{i,j-1})+\,\#({{\mathscr{S}}}_{i,j}\cap {{\mathscr{S}}}_{i,j+1})]\mathrm{.}$$

Here, ${{\mathscr{S}}}_{i,j}$ denotes the set of top *k* matches returned from the dictionary indexing run for the EBSD pattern at location (*i*, *j*) and # denotes the cardinality of a set. The orientations in the dictionary are selected to uniformly sample orientation space using the cubochoric representation^18^. The dictionary indexing technique has previously demonstrated superior performance for fast acquisition conditions in poly-crystalline Nickel^19^ as well as low symmetry geological materials^20^. Additional maps derived from the DI approach can be found in Figs 2–5 in the Supplementary Material.

The forward model fuses the deterministic Schrödinger equation, accounting for the elastic scattering of the electrons, with a Monte Carlo trajectory simulation to model the stochastic scattering^21^. The standard Bloch wave formalism is used to solve for the electron wave function inside the periodic crystal potential. The model computes the backscatter yield of the electron traveling in the direction $\hat{{\bf{k}}}$ with energy *E* as:

2 $${\mathscr{S}}(\hat{{\bf{k}}})=\sum _{{E}_{min}}^{{E}_{max}} {\mathcal E} (\hat{{\bf{k}}},E){\mathscr{P}}(\hat{{\bf{k}}},E,{z}_{0}(E)),$$

3 $${\mathscr{P}}(\hat{{\bf{k}}},E,{z}_{0}(E))=\sum _{n}\sum _{i\in {S}_{n}}\frac{{Z}_{n}^{2}{D}_{n}}{{z}_{0}(E)}{\int }_{0}^{{z}_{0}(E)}\lambda (E,z)|{\rm{\Psi }}({{\bf{r}}}_{i}){|}^{2}{\rm{d}}z\mathrm{.}$$

Here, ${\mathscr{S}}(\hat{{\bf{k}}})$ denotes the overall backscatter yield, Ψ(**r**_*i*_) denotes the wave function of the electron at the atomic site *i* and *λ*(*E*, *z*) denotes the depth and energy histogram derived from the Monte Carlo simulation; *S*_*n*_ is the set of equivalent positions, and *n* labels the unique positions in the asymmetric unit; *Z*_*n*_ and *D*_*n*_ are the atomic number and Debye-Waller factor, respectively, for the species at unique positio*n n*. The overall signal for an exit direction $\hat{{\bf{k}}}$ is an integration over the various energies contributing to the backscatter signal. This dynamical signal modulates a background signal, ${\mathcal E} (\hat{{\bf{k}}},E)$, and produces the characteristic Kikuchi bands. The stochastic part of the signal formation, including the angular histogram ${\mathcal E} (\hat{{\bf{k}}},E)$ and the energy and depth histogram *λ*(*E*, *z*), is determined using Monte Carlo electron trajectory simulations^22^.

For typical operating parameters, the dictionary indexing method has an accuracy of 0.7°; this error remains well under 1° for a range of errors in the geometrical calibration. The accuracy remains relatively constant in the presence of significant noise (≈45 dB peak signal to noise ratio) as well as a high camera binning factor $(\,\sim \,19\,\times \,)$^3^. In addition to regular indexing, the aforementioned forward model can also be used to perform a refinement step. This step searches the orientation space around the solution from the dictionary indexing to find a better match. The search is performed using the bobyQA (bound optimization by Quadratic Approximation) optimization algorithm^23^. This step is especially crucial for dictionary indexing of highly deformed materials to alleviate the artifacts introduced by the discrete sampling of orientation space. The orientation refinement step improves the accuracy of the method for well calibrated systems to $\sim 0.2^\circ$ for a range of noise levels for which the traditional Hough transform method fails to find a solution^24^.

### EBSD data analysis

All the EBSD data analysis was performed using the open source MTEX^25^ software. Grains were clustered using the default Voronoi tessellation method with a 15° threshold^26^. Only grains with areas larger than the threshold value of 0.08 *μm*^2^ were considered for the analysis. The discrete orientation distribution function (ODF) of the nanocrystalline zone was fitted to the symmetrized de la vallée Poussin kernel with a half-width of 10° to obtain the pole figures^27^. A similar method was used to calculate the correlated misorientation distribution function (MDF) of the grain boundaries.

### Data availability

The datasets generated and analysed during the current study are available from the corresponding author on reasonable request.

## Electronic supplementary material

Supplementary Information

## References

[1] Schwartz, A. J., Kumar, M., Adams, B. L. & Field, D. P. *Electron Backscatter Diffraction in Materials Science* (Springer, 2009).

[2] Chen YH (2015). A dictionary approach to EBSD indexing. Microscopy and Microanalysis.

[3] Ram F, Wright S, Singh S, De Graef M (2017). Error analysis of the crystal orientations obtained by the dictionary approach to EBSD indexing. Ultramicroscopy.

[4] Winiarski B (2016). High spatial resolution evaluation of residual stresses in shot peened specimens containing sharp and blunt notches by micro-hole drilling, micro-slot cutting and micro-x-ray diffraction methods. Experimental Mechanics.

[5] Altenberger I, Scholtes B, Martin U, Oettel H (1999). Cyclic deformation and near surface microstructures of shot peened or deep rolled austenitic stainless steel AISI 304. Materials Science and Engineering: A.

[6] Tao N, Sui M, Lu J, Lua K (1999). Surface nanocrystallization of iron induced by ultrasonic shot peening. Nanostructured Materials.

[7] Liu G, Lu J, Lu K (2000). Surface nanocrystallization of 316L stainless steel induced by ultrasonic shot peening. Materials Science and Engineering: A.

[8] Kamaya M, Wilkinson AJ, Titchmarsh JM (2006). Quantification of plastic strain of stainless steel and nickel alloy by electron backscatter diffraction. Acta Materialia.

[9] Child D, West G, Thomson R (2011). Assessment of surface hardening effects from shot peening on a ni-based alloy using electron backscatter diffraction techniques. Acta Materialia.

[10] Thomas, M. & Jackson, M. The role of temperature and alloy chemistry on subsurface deformation mechanisms during shot peening of titanium alloys. *Scripta Materialia* **66**, 1065–1068 Viewpoint Set no. 50: Twinning Induced PlasticitySteels (2012).

[11] Thomas M, Lindley T, Rugg D, Jackson M (2012). The effect of shot peening on the microstructure and properties of a near-alpha titanium alloy following high temperature exposure. Acta Materialia.

[12] Soady K (2013). Evaluating surface deformation and near surface strain hardening resulting from shot peening a tempered martensitic steel and application to low cycle fatigue. International Journal of Fatigue.

[13] Messé OMDM, Stekovic S, Hardy MC, Rae CMF (2014). Characterization of plastic deformation induced by shot-peening in a Ni-base superalloy. JOM.

[14] Fargas G, Roa J, Mateo A (2015). Effect of shot peening on metastable austenitic stainless steels. Materials Science and Engineering: A.

[15] Mackenzie J (1958). Second paper on the statistics associated with the random disorientation of cubes. Biometrika.

[16] Winiarski B, Burnett TL, Withers PJ (2016). Xe^+^ plasma FIB milling and lift-out approach for site-specific preparation of large volume blocks for 3D - EBSD. Microscopy and Microanalysis.

[17] Burnett T (2015). Large volume serial sectioning tomography by Xe plasma FIB dual beam microscopy. Ultramicroscopy.

[18] Singh S, De Graef M (2016). Orientation sampling for dictionary-based diffraction pattern indexing methods. Modeling and Simulation in Material Science and Engineering.

[19] Wright SI (2015). Introduction and comparison of new EBSD post-processing methodologies. Ultramicroscopy.

[20] Marquardt K (2017). Quantitative electron backscatter diffraction (EBSD) data analyses using the dictionary indexing (DI) approach: Overcoming indexing difficulties on geological materials. American Mineralogist.

[21] Callahan PG, De Graef M (2013). Dynamical electron backscatter diffraction patterns. part i: Pattern simulations. Microscopy and Microanalysis.

[22] Joy, D. C. *Monte Carlo Modeling for Electron Microscopy and Microanalysis* (Oxford University Press, 1995).

[23] Powell, M. The BOBYQA algorithm for bound constrained optimization without derivatives. Tech. Rep., Department of Applied Mathematics and Theoretical Physics, Cambridge University (2009).

[24] Singh, S., Ram, F. & De Graef, M. Application of forward models to crystal orientation refinement. *Journal of Applied Crystallography* **50** (2017).

[25] Bachmann, F., Hielscher, R. & Schaeben, H. Texture analysis with MTEX – free and open source software toolbox. In *Texture and Anisotropy of Polycrystals III*, vol. 160 of *Solid State Phenomena*, 63–68 (Trans Tech Publications, 2010).

[26] Bachmann F, Hielscher R, Schaeben H (2011). Grain detection from 2D and 3D EBSD data—specification of the MTEX algorithm. Ultramicroscopy.

[27] Hielscher R, Schaeben H (2008). A novel pole figure inversion method: specification of the MTEX algorithm. Journal of Applied Crystallography.

