# Supplementary material for: High resolution low kV EBSD of heavily deformed and nanocrystalline Aluminium by dictionary-based indexing
Source: Sci Rep. 2018 Jul 20;8:10991. doi: 10.1038/s41598-018-29315-8 (PMC6054629; doi:10.1038/s41598-018-29315-8)
Supplement: Supplementary file 1 — Supplementary Information [file 41598_2018_29315_MOESM1_ESM.pdf]

High resolution low kV EBSD of heavily deformed and nanocrystalline Aluminium by dictionary-based indexing Supplementary Information

Saransh Singh[1], Yi Guo[2], Bartlomiej Winiarski[2,3], Timothy L. Burnett[2,*], Philip J. Withers[2], and Marc De Graef[1,*]

[1]Carnegie Mellon University, Department of Materials Science and Engineering, Pittsburgh, PA15213-3890, USA
[2]School of Materials, University of Manchester, M13 9PL, UK
[3]Thermo Fisher Scientific, Materials and Structural Analysis Division, Brno, 627 00, Czech Republic
[*]timothy.burnett@manchester.ac.uk, degraef@cmu.edu

July 1, 2018

Supplementary Information

Background

Venables and Harland recorded the first electron backscatter diffraction images in 1973 [1] and the first practical system was developed by D. Dingley and Link in 1987. A commercial system (HKL) arrived in 1990. This enabled the direct visualization of the individual grains and mapping of their orientations. Recent years have seen the emergence of high energy diffraction microscopy (HEDM), which uses a synchrotron source to map grain orientations in 3-D [2], and grains can even be mapped in the laboratory by diffraction contrast tomography with excellent angular resolution, although at inferior spatial resolution [3]. At higher resolutions, precession electron diffraction (PED) can be carried out in a transmission electron microscope (TEM) [4]. While the HEDM technique can gather 3-D data from millimeter scale samples very quickly, it lacks the spatial resolution to capture the nanometer scale features close to the peening surface. TEM-based techniques and the SEM-based TKD modality have very high spatial resolution, but are limited to electron transparent samples [5].

All of today's commercially available EBSD systems implement a variant of the Hough transform indexing approach. While the spatial and angular resolutions of this approach are strongly correlated with the microscope acquisition conditions as well as the material system under investigation, they are routinely quoted as $\sim$ 50 nm and $< 1^\circ$, respectively. These commercial techniques have been very successful in characterizing a wide range of material systems but they largely fail for highly deformed and/or small grain microstructures, which are often cases of particular interest. There are a number of reasons for this failure: firstly, the current design of the EBSD camera consists of a scintillator screen, which is optically connected through a fibre-optic bundle or lens to a charge coupled device (CCD) camera sitting behind the scintillator. The conversion of electrons to photons by the scintillator requires a minimum acceleration potential, so that a lower microscope acceleration voltage leads to significant loss in the definition of the Kikuchi bands. Secondly, the stored deformation in highly deformed materials leads to additional noise in the diffraction patterns. The result is that diffraction patterns of highly deformed materials obtained at a low acceleration voltage are largely unindexable by the current algorithms based on feature detection using the Hough transform. In the main document, a new technique is described that is capable of indexing diffraction patterns from highly deformed materials at low accelerating voltages.

Sample Preparation

Fig. 1 shows the sequence of steps followed to create the sample used for this study. The extraction of the block and the sectioning and polishing of the block face were conducted using a Thermo Fisher Scientific Helios^{TM} Plasma Xe^{+} FIB-SEM DualBeam equipped with EasyLift^{TM} in-situ nanomanipulator on a TEM grid attached to a pre-tilted EBSD sample holder.

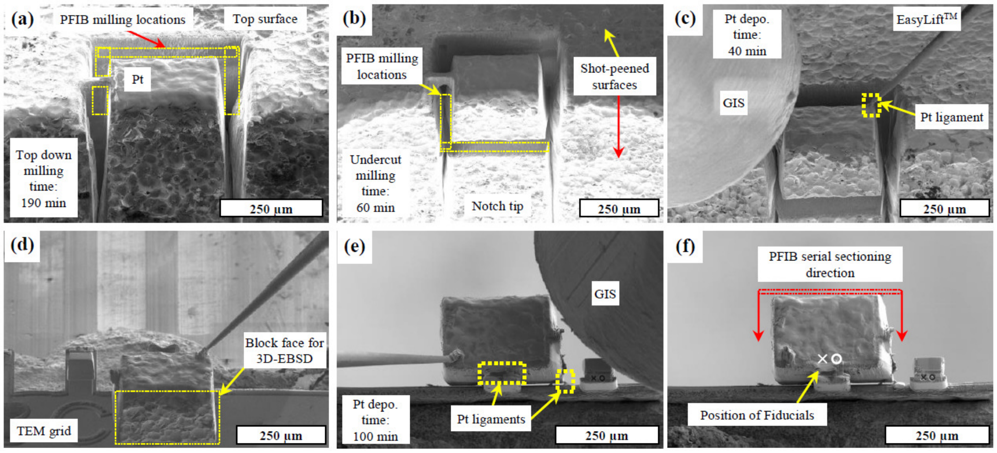

Figure 1: Details of the sample preparation process. Illustration of site-specific sample block preparation [6] of shot-peened 7075 with a notch (a-b) and lift-out procedure (c-f). (a, b, e, f) SEM images (5 kV @ 1.4 nA ETD SE); (c, d) PFIB images (30 kV @ 24 pA, ICE detector).

Example Experimental and Simulated Patterns

Fig. 2 shows three experimental raw EBSD patterns (top row) taken from a small grain in the dynamically recrystallized region near the shot peen crater (a), in the heavily deformed region about 20 μm from the crater (c), and about 45 μm from the crater (e) in the region that is not strongly deformed. The second row shows dynamically simulated patterns for the orientations produced by the dictionary indexing algorithm followed by orientation refinement. The best matching dot product value is shown at the bottom; these values should be compared to the value of 0.82, which is the average value for non-matching dictionary patterns. While the dot product values decrease when approaching the shot peen crater, they remain significantly larger than the value for non-matches, indicating that the DI technique has no issues indexing all the experimental patterns. The orientation Euler angle triplets for the three patterns are $(32.23°, 35.38°, 355.83°)$ for pattern (a), $(115.70°, 18.44°, 244.25°)$ for pattern (c), and $(242.41°, 3.86°, 127.03°)$ for pattern (e).

Additional Indexing Results

The Dictionary Indexing (DI) computations were executed using a series of open source programs from the EMsoft package, available from URL: `https://github.com/marcdegraef/EMsoft`. The sequence of programs is as follows:

1. EMmkxtal: generates a crystal structure file in HDF5 format, containing lattice parameters, space group number, atom coordinates for all atoms in the asymmetric unit, site occupation parameters and Debye-Waller parameters.

2. EMMCOpenCL: computes energy, direction, and depth histograms using a GPU-OpenCL Monte Carlo implementation based on the continuous slowing down approximation.

3. EMEBSDmaster: combines the Monte Carlo output with a dynamical scattering computation to generate an EBSD master pattern from which individual patterns can be computed using bilinear interpolation.

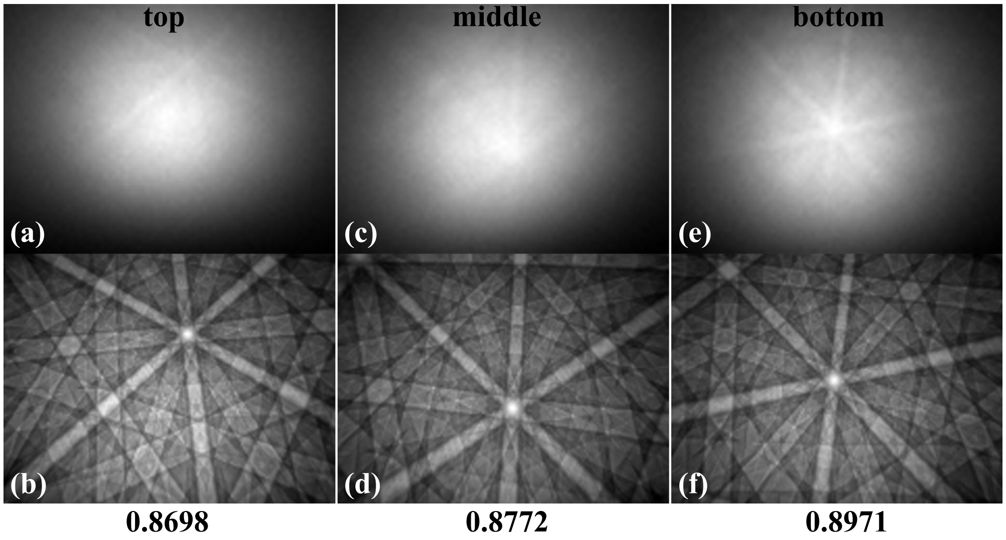

Figure 2: Examples of experimental and simulated EBSD patterns. Experimental EBSD patterns (top row) from three locations in the region of interest (see text for details); simulated patterns are shown in the second row, and the best matching (refined) dot product value is shown at the bottom.

4. EMEBSDDI: the dictionary indexing program takes as input the set of experimental patterns along with the detector parameters, and performs the matching process using the dot product between normalized vectors; this program produces both an HDF5 output file and a pair of Oxford format .ctf files that can be read by other EBSD programs, such as the Aztec commercial package and the open source MTEX package.

5. EMFitOrientation: this is a refinement program that starts with the highest dot product orientation of the previous step and refines the orientation to maximize the dot product between experimental and dictionary patterns; the output is a .ctf file.

In addition to the Orientation Similarity Map defined in the Methods section of the main paper and shown in Fig. 2b, the DI approach generates a number of additional maps that are useful for the study of this highly deformed microstructure; the following subsections provide additional details.

Average Dot Product Map

The raw experimental data can be used to generate a so-called Average Dot Product (ADP) map; after background subtraction and adaptive histogram equalization, each EBSD pattern is normalized into a unit-length column vector, and the average dot product is computed between each pattern and its four nearest neighbors (corrected for edge and corner patterns). The ADP map, shown in Fig. 3, has a high intensity in regions where the EBSD patterns of nearest neighbor pixels are very similar (in this case near the bottom of the map, in the less distorted regions). Near grain boundaries, or in the heavily distorted region, the ADP value drops significantly; thus, the ADP map is an indicator of the level of distortion in the microstructure. The brighter region at the top of the map is a protective platinum capping layer.

Image Quality Map

The commercial software packages define the pattern quality by means of the average height of the detected peaks in the pattern's Hough transform. The DI approach, on the other hand, does not extract the bands from the pattern, so an alternative definition of the pattern (or image) quality is needed. We have used the pattern sharpness parameter Q introduced by Lassen [7], which is defined as:

$$Q = 1 - \frac{J}{J_{\text{res}} w_{\text{tot}}}, \tag{1}$$

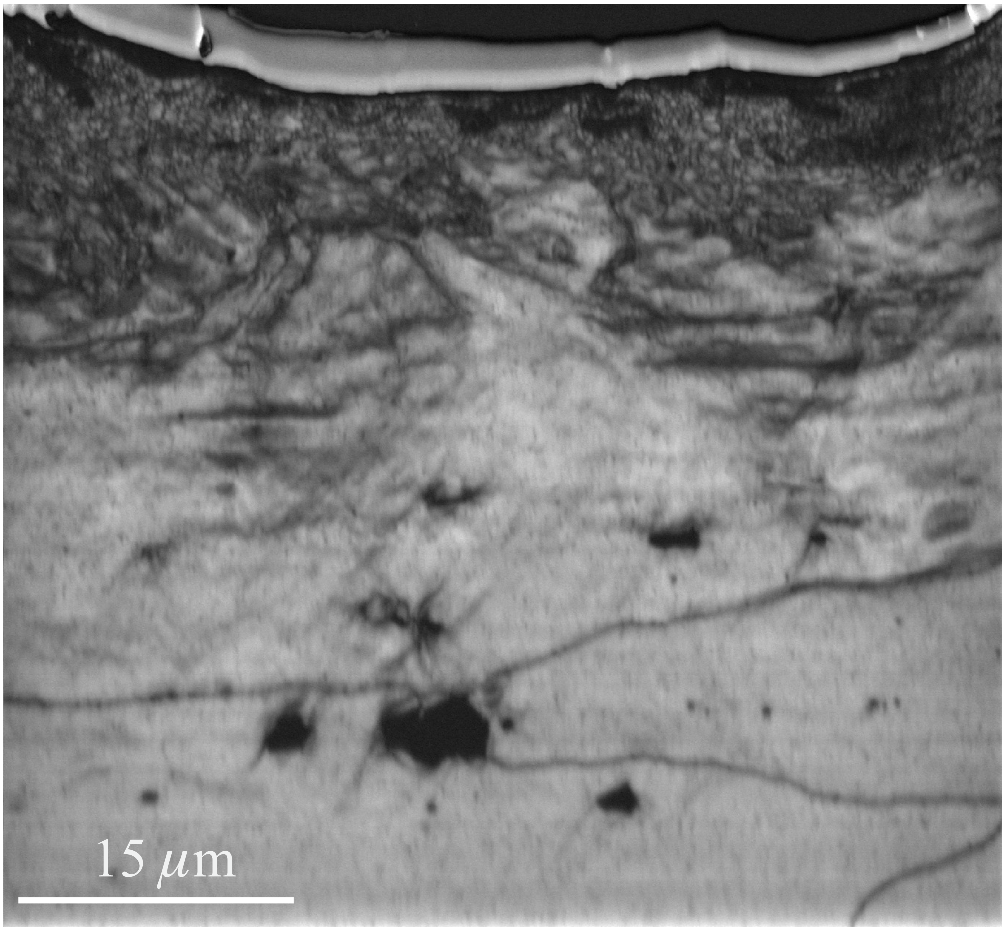

Figure 3: Average dot product map. Average dot product map computed from the raw 10kV EBSD patterns; the field of view measures 56×52 μm^2.

where

$$J = \sum_{h,k=-N'}^{N'} w(h,k)(\mathbf{q})^2, \quad J_{\text{res}} = \frac{1}{N^2} \sum_{h,k=-N'}^{N'} |\mathbf{q}|^2, \quad \text{and} \quad w_{\text{tot}} = \sum_{h,k=-N'}^{N'} w(h,k), \tag{2}$$

with $\mathbf{q} = (h,k)$ the frequency vector, $w(h,k)$ the power spectrum of the original EBSD pattern, and $N' = N/2$ with N the pattern size (for a square $N \times N$ pattern). The IQ map for the present data set is shown in Fig. 4. The map essentially measures how far out in Fourier space the pattern information is present; for well defined patterns, the Q value is high, whereas in the deformed regions the value decreases. Note that the amorphous (or nano-crystalline) Pt cap does not produce EBSD patterns at all.

Confidence Index Map

The confidence index informs about the reliability of the indexing result for each individual pattern. In the commercial packages, this index is computed based on the number of Kikuchi bands that are properly reproduced by the various indexing solutions; the index is the difference in votes received between the highest and second highest-ranking solutions, divided by the total number of possible votes. In the DI approach, the Confidence Index is taken to be the highest value dot product, i.e., the similarity between the experimental pattern and the best matching dictionary solution. This index also serves as the parameter that is maximized during the indexing refinement step. The CI map for the present microstructure is shown in Fig. 5. Note how the CI values remain high (bright) even in the recrystallized zone, clearly delineating the individual

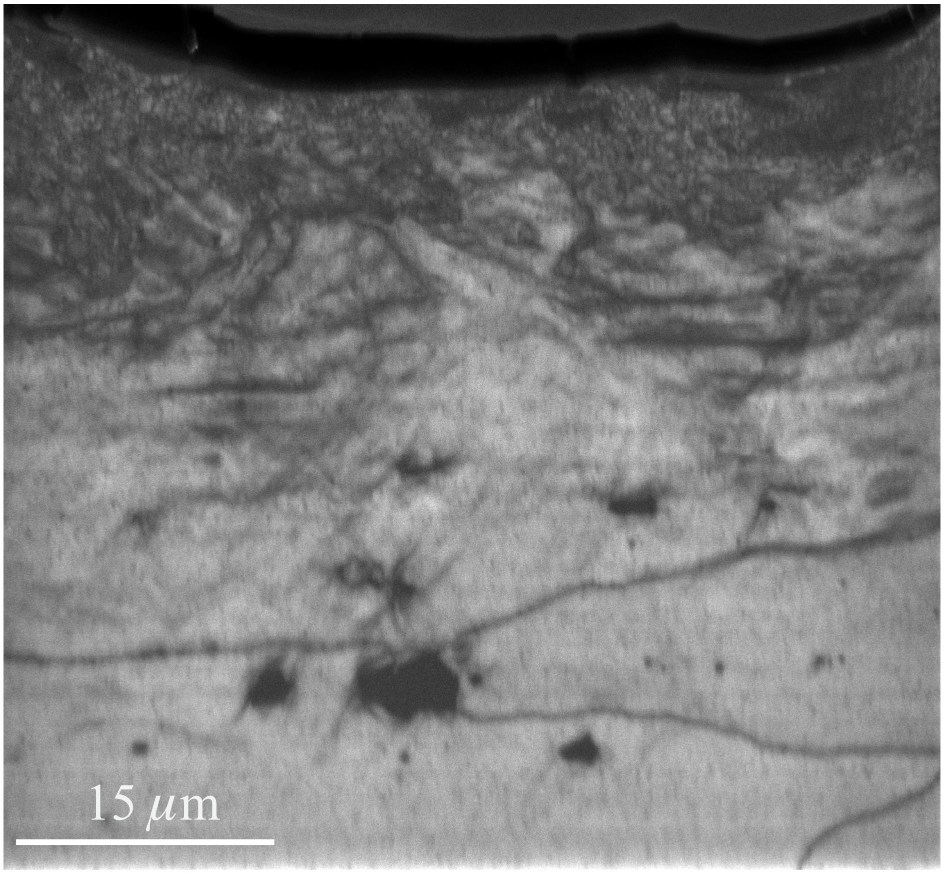

Figure 4: Image Quality map. Image Quality map computed from the raw 10kV EBSD patterns.

nano-crystalline grains. This clear delineation of the smallest grains is also visible in the magnified portion of the OSM map shown in Fig. 6. The smallest grains are of the order of 100 nm in size.

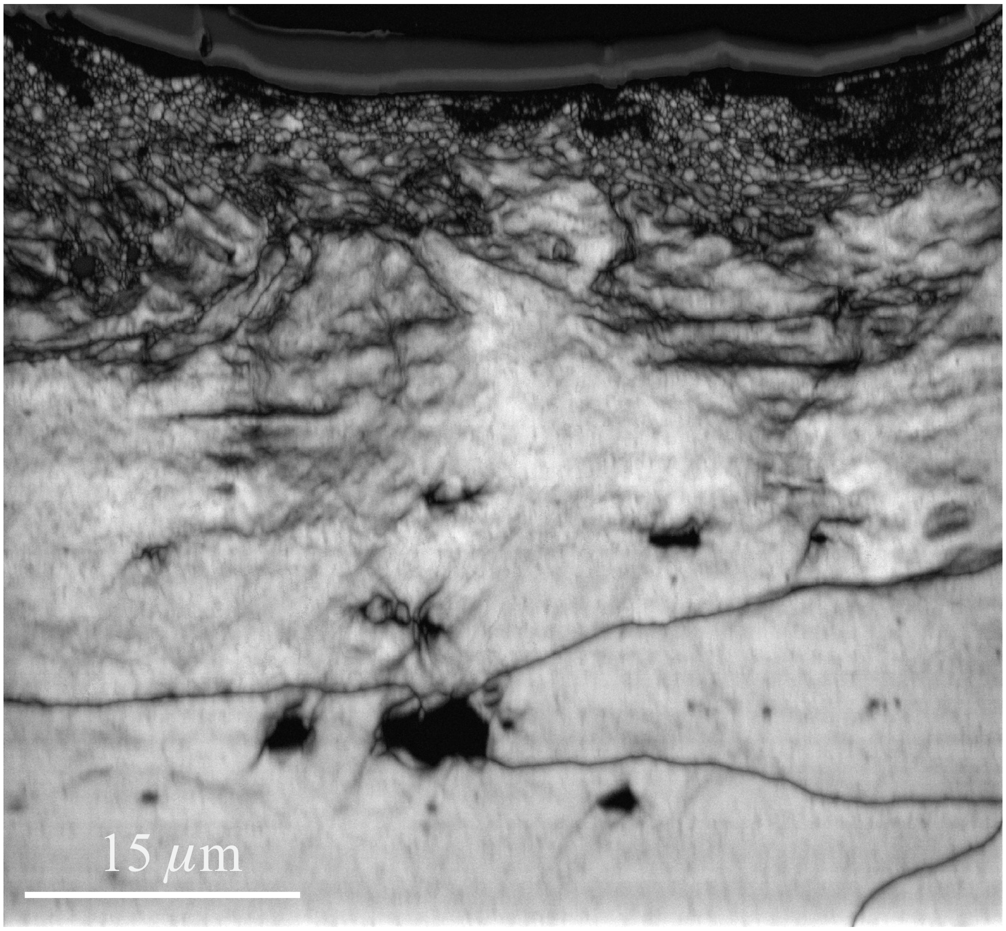

Figure 5: Confidence Index map. Confidence Index map after refinement of the orientation obtained with the dictionary indexing algorithm.

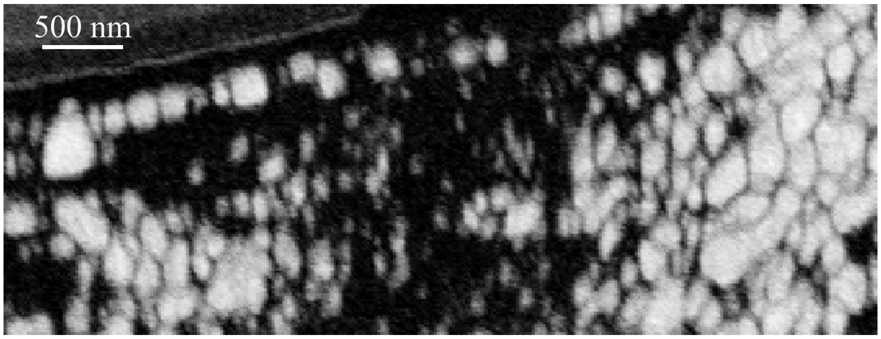

Figure 6: Orientation Similarity Map segment. Small section from the OSM map of Fig. 2b of the main paper, showing the nano-size recrystallized grains at the base of the shot peen crater.

Additional Inverse Pole Figure Maps

Figs. 7-9 show the complete IPF maps for the entire field of view of $56 \times 52\ \mu m^2$, for the (100), (010), and (001) sample directions, respectively.

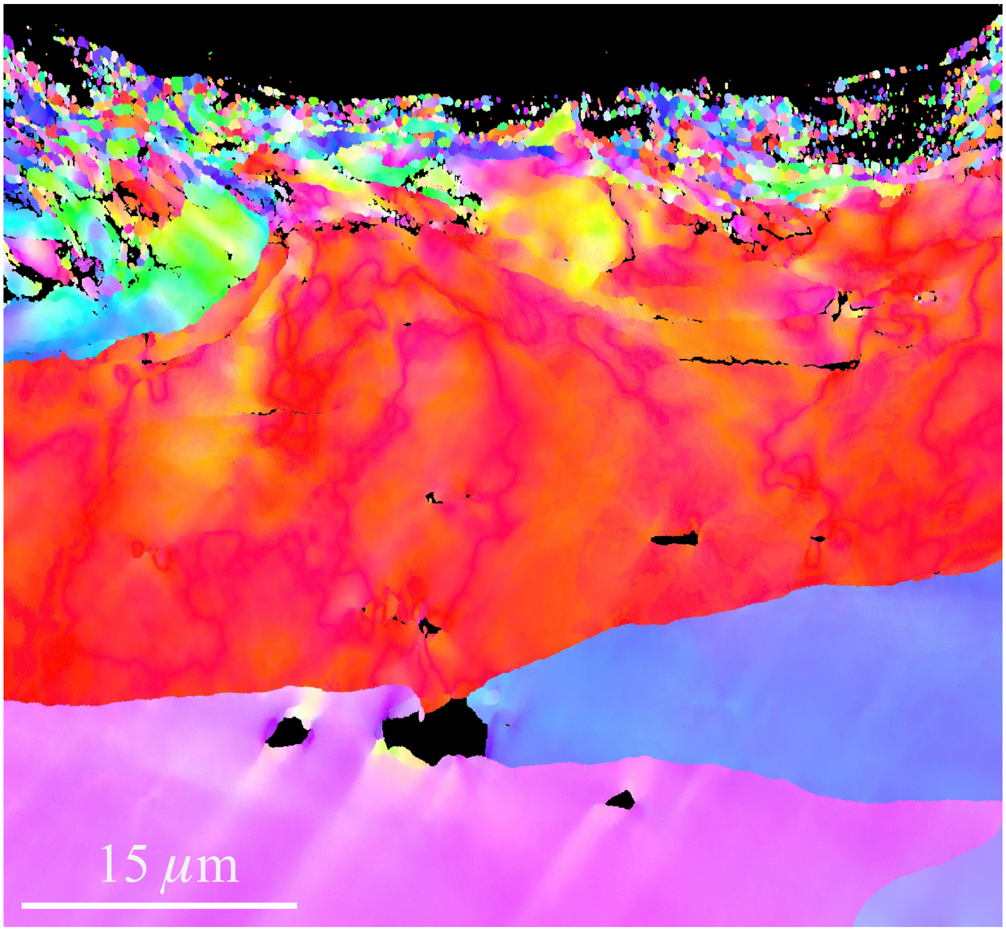

Figure 7: (100) inverse pole figure map after orientation refinement.

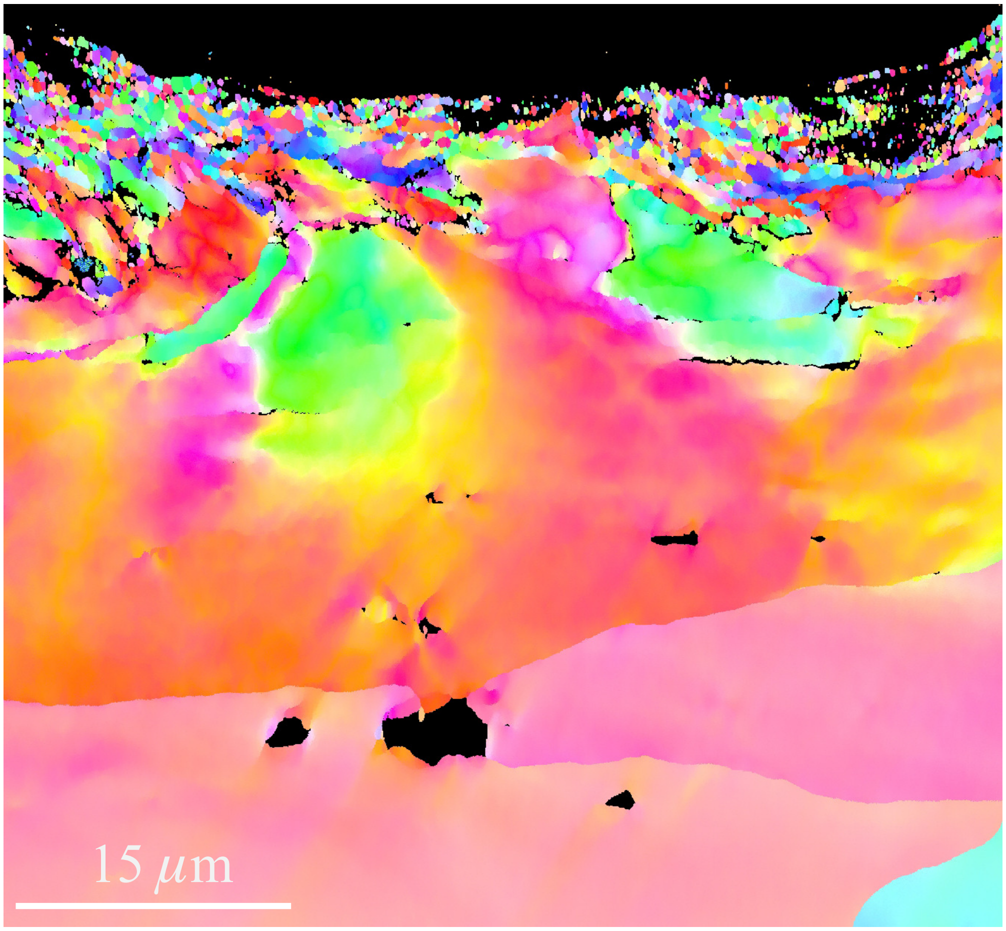

Figure 8: (010) inverse pole figure map after orientation refinement.

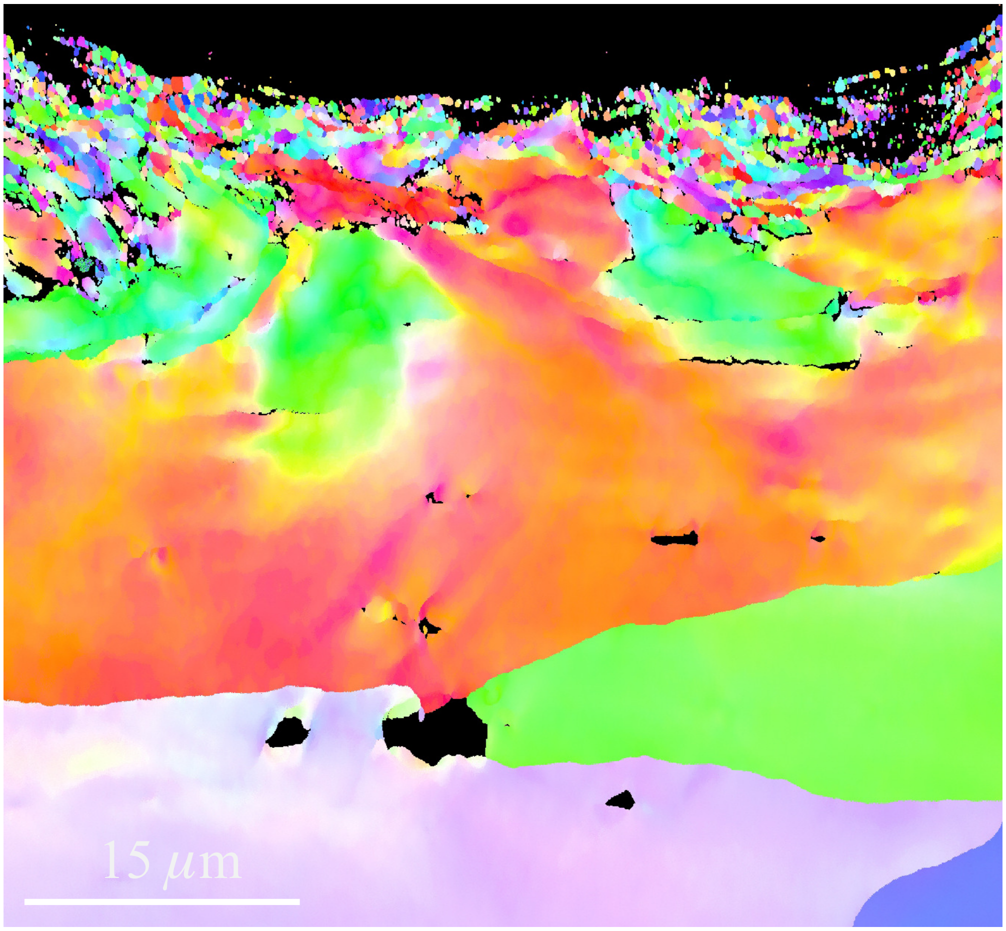

Figure 9: (001) inverse pole figure map after orientation refinement.

References

[1] Venables, J. & Harland, C. Electron back-scattering patterns – a new technique for obtaining crystallographic information in the scanning electron microscope. *Phil. Mag.* **27**, 1193–1200 (1973).

[2] Poulsen, H. F. *et al.* 3-dimensional characterization of polycrystalline bulk materials using high-energy synchrotron radiation. In *THERMEC 2006*, vol. 539 of *Materials Science Forum*, 2353–2358 (Trans Tech Publications, 2007).

[3] McDonald, S. *et al.* Microstructural evolution during sintering of copper particles studied by laboratory diffraction contrast tomography (LabDCT). *Scientific Reports* **7**, 5251 (2017).

[4] Rauch, E. F. & Duft, A. Orientation maps derived from TEM diffraction patterns collected with an external CCD camera. In *Textures of Materials - ICOTOM 14*, vol. 495 of *Materials Science Forum*, 197–202 (Trans Tech Publications, 2005).

[5] Trimby, P. W. Orientation mapping of nanostructured materials using transmission kikuchi diffraction in the scanning electron microscope. *Ultramicroscopy* **120**, 1624 (2012).

[6] Winiarski, B., Burnett, T. L. & Withers, P. J. Xe^+ plasma FIB milling and lift-out approach for site-specific preparation of large volume blocks for 3D - EBSD. *Microscopy and Microanalysis* **22**, 838839 (2016).

[7] Krieger Lassen, N., Juul Jensen, D. & Conradsen, K. On the statistical analysis of orientation data. *Acta Crystallographica A* **50**, 741748 (1994).
